# Relevance of TRPA1 and TRPM8 channels as vascular sensors of cold in the cutaneous microvasculature

**DOI:** 10.1007/s00424-017-2085-9

**Published:** 2017-11-21

**Authors:** Y. Pan, D. Thapa, L. Baldissera, F. Argunhan, A. A. Aubdool, S. D. Brain

**Affiliations:** Section of Vascular Biology and Inflammation, School of Cardiovascular Medicine and Research, BHF Cardiovascular Centre of Research Excellence, King’s College London, Room 3.74, FWB, 150 Stamford Street, London, SE1 9NH UK

**Keywords:** TRPA1, TRPM8, Cold, Vascular, Blood flow, Thermoreceptors

## Abstract

Cold exposure is directly related to skin conditions, such as frostbite. This is due to the cold exposure inducing a vasoconstriction to reduce cutaneous blood flow and protect against heat loss. However, a long-term constriction will cause ischaemia and potentially irreversible damage. We have developed techniques to elucidate the mechanisms of the vascular cold response. We focused on two ligand-gated transient receptor potential (TRP) channels, namely, the established “cold sensors” TRP ankyrin 1 (TRPA1) and TRP melastin (TRPM8). We used the anaesthetised mouse and measured cutaneous blood flow by laser speckle imaging. Two cold treatments were used. A generalised cold treatment was achieved through whole paw water immersion (10 °C for 5 min) and a localised cold treatment that will be potentially easier to translate to human studies was carried out on the mouse paw with a copper cold probe (0.85-cm diameter). The results show that TRPA1 and TRPM8 can each act as a vascular cold sensor to mediate the vasoconstrictor component of whole paw cooling as expected from our previous research. However, the local cooling-induced responses were only blocked when the TRPA1 and TRPM8 antagonists were given simultaneously. This suggests that this localised cold probe response requires both functional TRPA1 and TRPM8.

## Introduction

### The vascular response

When the skin surface is exposed to cold, the underlying blood vessels contract to prevent heat loss by restricting the blood flow. This is normally a transient event, to prevent the cell/tissue death (e.g. in the event of frostbite) from ischaemia. The blood vessels subsequently dilate to resume blood flow and perfuse the tissue for protection and survival. This basic adaptive physiological mechanism of vasoconstriction followed by vasodilation was first described by Thomas Lewis [35]. If one ‘fast forwards’ almost 90 years with substantial research carried out, today, we have a much better understanding of the phenomenon [12, 28]. We have developed a system whereby a mouse was anaesthetised and the paw immersed up until a depth of approximately 1.5 cm in cold water (10 °C for 5 min). The blood flow response was monitored before and then after immersion and a response that is detailed below was found. From this study, we now know that transient receptor potential channel TRPA1 plays a key role as a vascular cold sensor and that TRPM8 is also involved [4]. However, the mechanisms involved in the vascular cold response are not fully understood [13, 36].

### “Cold” receptors: TRPA1 and TRPM8

#### TRPA1

TRPA1 was discovered in 1999 and is the sole member of the TRPA1 family [24]. It belongs to the superfamily of TRP receptor and is a ligand-gated cation channel. TRPA1 is primarily found in sensory neurons where it is highly co-expressed (60–70%) with TRP vanilloid 1 (TRPV1) channels, which was discovered in 2003 by Story and colleagues [44]. TRPA1 is known for its sensory function in detection of noxious cold and is additionally activated by a range of chemicals including vegetable extracts such as allyl isothiocyanate, mustard oil, and allicin as well as electrophiles [20]. The first evidence for a role of TRPA1 in sensing cold was discovered when Chinese hamster ovary (CHO) cells expressing TRPA1 receptors were shown to be activated by cold (< 17 °C) [44]. The study showed that cold buffer induced a rise in calcium ions in TRPA1-expressing CHO cells but not in non-transfected CHO cells. Additionally, higher temperature (20–37 °C) did not activate TRPA1 which strongly suggested that TRPA1 is a cold-specific channel. Following on from that study, several other independent groups including ours have produced data supporting the role of TRPA1 in sensing cold, usually relating it to pain sensation [2, 7, 9, 17, 22, 27, 29, 32]; although, our own recent research has concentrated on the role of TRPA1 as a vascular cold sensor [5].

#### TRPM8

TRPM8 was discovered as mRNA, which was upregulated in prostate cancer [46]. However, it was later identified as a TRP channel activated by cold ([37, 41]. TRPM8 is a non-selective cation channel and similar to TRPA1, it is expressed in a distinct subset of sensory nerves that have a different profile to TRPA1-containing sensory nerves [41]. Belonging to the sub-family of TRPM, TRPM8 is activated by cool temperatures in the range of 10–28 °C and by chemical agents including menthol, icilin, and several inflammatory agents. In 2007, TRPM8 KO mice were shown to lack cold sensation, cold allodynia, and analgesia which confirmed the role of TRPM8 in cool sensation [10, 15, 18]. Although TRPM8 is well established to be responsible for sensing innocuous cold, there is evidence in the literature which suggest that it may additionally play a role in sensing painful/nociceptive cold [10, 40, 48] and deep body cooling [16].

#### TRPA1, TRPM8, and the vascular responses

TRPA1 can influence vascular tone. The first evidence came from Bautista, who, using the TRPA1 agonist allicin, revealed that activation of TRPA1 channel on capsaicin-sensitive peptidergic nerve fibres induces vasodilation of the mesenteric artery [9]. Using TRPA1 KO mice, Pozsgai showed that TRPA1 causes cardiovascular effects by influencing some cardiovascular effects [42] but this was difficult to define in terms of relevance to cardiovascular disease using TRPA1 knockout mice [11]. Furthermore, TRPA1 has been shown to play a central role in mediating blood flow in the cerebral circulation [21] and, mouse paw and ear [6, 23].

Likewise, TRPM8 has been shown to play a role in vascular tone. It can stimulate both vasoconstriction and vasodilation depending on the previous vasomotor tone of the blood vessel [25]. The same study also showed that topical application of menthol causes an increased blood flow in conscious humans. Menthol provides a cold sensation in the human skin, and this has been shown to be associated with an increased blood flow in the skin, although it is unknown as yet whether this is TRPM8 dependent [31]. A study by Sun and colleagues showed that activation of TRPM8 attenuated vasoconstriction of the mesenteric artery and lowered blood pressure via the RhoA/Rho kinase pathway. The authors proposed its agonist menthol as a potential therapeutic diet for hypertensive patients [45]. Hence, the role of TRPA1 and TRPM8 in controlling the vascular tone is well established, but, it was the recent extensive study from our group that revealed the mechanisms via which TRPA1/TRPM8 mediates vascular tone in response to the cold [5].

Our study showed that TRPA1 is an essential vascular sensor of cold, playing a role in both vasoconstriction and the subsequent vasodilation [5]. The study showed that TRPA1 initiates the vascular response to cold by causing vasoconstriction, as this constrictor response was absent in the presence of TRPA1 antagonist and TRPA1 KO mice. TRPM8 was also shown to be involved in the process as the response was partially but significantly blunted with TRPM8 antagonist. The response was revealed to be mediated via Rho-kinase-mediated MLC phosphorylation downstream of α_2c_-adrenoceptors following superoxide generation. Additionally, the study revealed that TRPA1 also plays an important role in vasodilation and restoration of blood flow. TRPA1 was shown to mediate vasodilation via sensory nerve-derived neuropeptides calcitonin gene-related peptide (CGRP) and substance P, and neuronal nitric oxide (nNOS)-derived NO. Altogether, we demonstrated that TRPA1 plays a fundamental role in mediating vascular response to cold.

Here, we have extended our investigation into the role of TRPA1 and TRPM8 in the noxious cold-induced vascular response. Specifically, we aim to understand how TRPA1 and TRPM8 affect the blood flow under localised cold (using a copper cold probe) in comparison to generalised cold (water immersion) conditions in the mouse paw.

## Methods

### Animals

Male CD1 mice (7–9 weeks of age from Charles River, UK) were used in all experiments. Mice were housed in a climatically controlled environment, with free access to water and food, on a 12-h light/dark cycle. Experiments were conducted in accordance with the UK Home Office Animals (Scientific Procedures) Act, 1986 and were approved by the King’s College London Animal Care and Ethics Committee. Animals were randomly assigned to control or treatment groups, and experiments were conducted in a blinded manner.

### The vascular cold response

Two techniques were used to induce cold exposure: water immersion technique and a cold copper probe (0.85-cm diameter). The copper probe attached to a thermos cup which is filled with 20% sodium chloride and stored in − 20 °C freezer overnight to get the temperature of probe at 10 °C. To measure the cold-induced vascular response, mice were anaesthetised (i*.*p.) with ketamine (75 mg/kg) plus medetomidine (1 mg/kg), placed on a heating mat in ventral position to maintain the body temperature at 36.5 °C, and with room temperature kept at 24 ± 2 °C. The hind paw cutaneous blood circulation was monitored and measured by the full-field laser perfusion imager (FLPI, Moor Instruments, UK) as described in [5]. The blood flow of the paws and tail was assessed for 5 min using FLPI first as the baseline, then the ipsilateral paw was immersed in ice water at 10 °C (generalised cooling) or touched with cold copper probe (local cooling) at 10 °C for 5 min, while the contralateral paw and tail received no treatments and were observed as controls. A traditional thermometer was immersed in the cold ice water to monitor and adjust the temperature, whereas the temperature of the cold probe was measured using an infrared thermometer (ThermoWorks TW2). After the cold exposure, the blood flow was recorded for the next 30 min. Due to technical limitations, the blood flow change during the actual cold exposure period could not be measured.

### Antagonists and inhibitors

The TRPA1 antagonist HC030031 (2-(1,3-dimethyl-2,6-dioxo-1,2,3,6-tetrahydro-purin-7yl)-*N-*(4-isopropyl-phenyl)-acetamide 1) and TRPM8 antagonist AMTB (*N*-(3-aminopropyl)-2-[(3-methylphenyl) methyl] oxy-*N*-(2-thienylmethyl) benzamide hydrochloride salt) were purchased from Tocris and Sigma, respectively. Both drugs were dissolved in 10% DMSO in saline and administered (i.p.) at a dose of 100 mg/kg for HC030031 and 10 mg/kg for AMTB 30 min before the cold exposure [3, 5, 34].

### Statistical analysis

The data calculation was carried out using Microsoft excel and statistical analysis was done using Graphpad Prism 5. Results are expressed as mean ± SEM, and *p* < 0.05 was considered to be significant. Analysis of variance with Bonferroni post hoc test was used to calculate significant difference.

## Results

### The comparison of the vascular response to generalised and localised cold

**Fig. 1:**
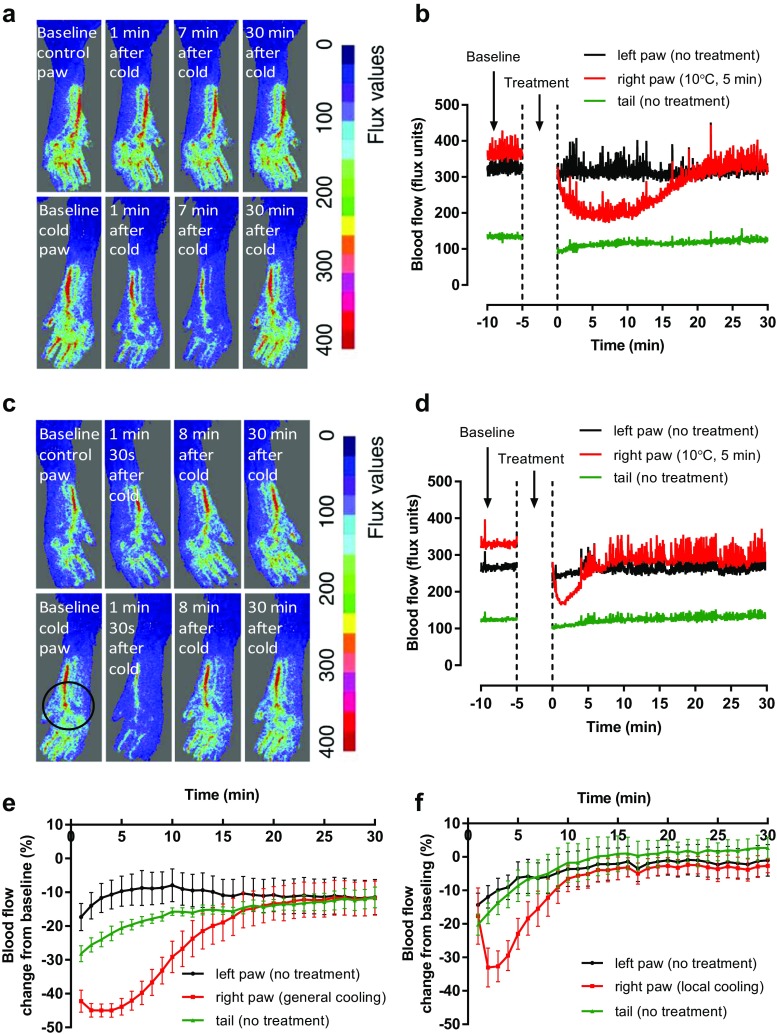
The effect of blood flow changes induced by 10 °C 5-min generalised cooling (water immersion) or local cooling (0.85-cm-diameter cold probe in mouse paw). Blood flow was monitored dynamically using FLPI in anaesthetized mice following ipsilateral (right) hind paw cooling. **a** Representative images of control (untreated) and cold paw before (baseline) and after 10 °C, 5-min water immersion. The blood flow is colour coded using a scale ranging from dark blue (low) to red (high). **b** Representative blood flow trace of the water immersion vascular response. **c** Representative images of control (untreated) and cold paw before (baseline) and after 10 °C local cold probe touch (circle highlights the area of cold probe touch). **d** Representative blood flow trace of the cold probe touch vascular response. **e** Mean blood flow data for cold water immersion for 0–30 min after cooling (*n* = 6). **f** Mean blood flow data for 0–30 min after cold probe (*n* = 5)

The representative images of the vascular response induced by two distinct cold techniques are shown in Fig. 1a–d. Before the cold treatment, blood flow in hind paws was maintained at around 300–400 flux units (baseline). Following the baseline measurement, the ipsilateral hind paw was immersed in cold water (10 °C for 5 min), while the contralateral hind paw remained untreated at room temperature (24 ± 2 °C). The cold water treatment resulted in vasoconstriction as shown by the reduction in blood flow, whereas the blood flow in the untreated contralateral paw remained unchanged (Fig. 1a, b). The maximum vasoconstriction is illustrated as percent maximum reduction in blood flow from the baseline and was observed 7 min after the end of generalised cooling, after which it slowly recovered back to the baseline. The cold copper probe produced a similar response with decreased blood flow in the ipsilateral hind paw, albeit a weaker one, suggesting cold probe induces less vasoconstriction compared to that of the water immersion technique (Fig. 1c, d). Interestingly, the cold probe induced maximum vasoconstriction approximately 2 min after the end of cold treatment and resulted in faster recovery of the blood flow back to the baseline (Fig. 1e, f). The result suggests that the two separate cold treatment techniques might produce vascular response via different mechanisms.

### Influence of TRPA1 and TRPM8 antagonists on the vascular response to cold

**Fig. 2:**
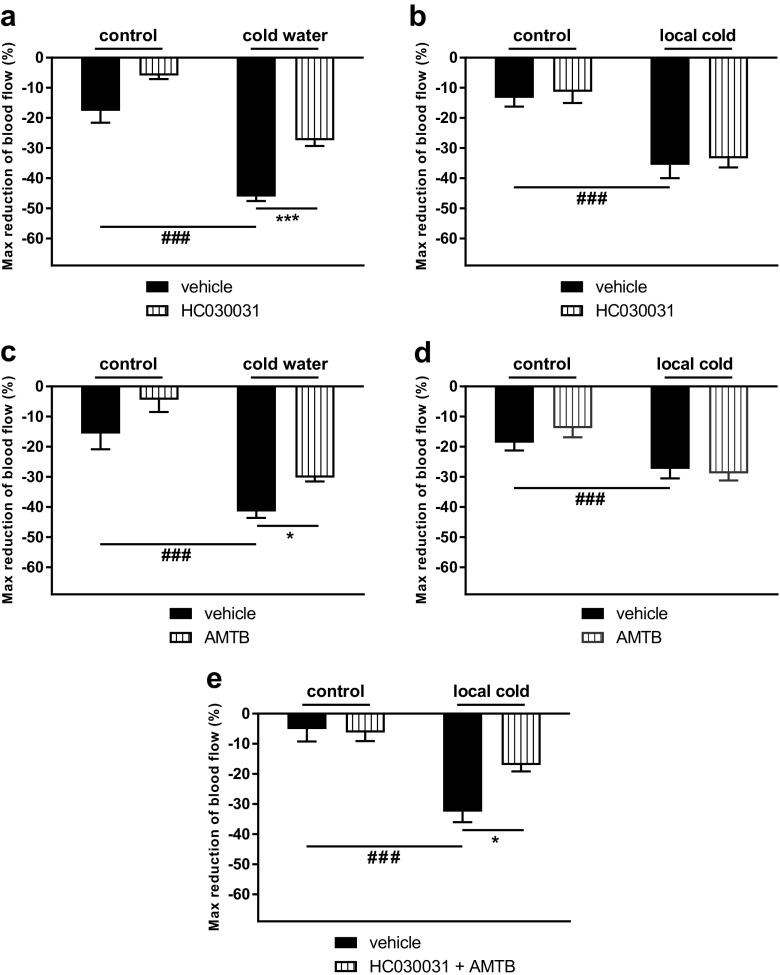
Graph shows percent maximum reduction in blood flow from baseline after cold treatment in mouse paw. **a** General cooling (water immersion) with HC03001 treatment (*n* = 6). **b** Local cooling (copper probe) with HC030031 treatment (*n* = 5). **c** General cooling (water immersion) with AMTB treatment (*n* = 6). **d** Local cooling (copper probe) with AMTB treatment (*n* = 7). **e** Local cooling (cold probe) with combined HC030031 and AMTB treatment (*n* = 5). Results are expressed as mean ± SEM and analysed by two-way ANOVA with Bonferroni post hoc test. *p < 0.05, **p < 0.01, ***p < 0.001, ###*p* < 0.001

To elucidate the differences in the cold-induced vascular response between the water immersion and cold probe techniques, we investigated the role of TRPA1 and TRPM8 channels. The TRPA1 antagonist HC030031 and TRPM8 antagonist AMTB were administered (i.p.) 30 min prior to the cold treatment and blood flow was measured using FLPI as described previously [5]. HC030031 significantly inhibited the vasoconstrictor response induced by cold water immersion but not by the cold probe (Fig. 2a, b). The result suggests that the TRPA1 channel plays a role in vascular response that is induced by the whole paw water immersion or generalised cooling but not by cold copper probe or localised cooling. It is possible that the thermo-sensors vary depending on the type of cold stimuli, which indicates a different thermoreceptor other than TRPA1 such as TRPM8 or an unknown thermochannel yet to be discovered is involved in the localised cooling. To investigate this, we used TRPM8 antagonist AMTB. AMTB significantly inhibited the vasoconstriction induced by the cold water immersion technique; however, similar to HC030031, AMTB did not affect vasoconstriction induced by the cold probe (Fig. 2c, d). This means neither TRPA1 nor TRPM8 can act alone to influence the vascular response mediated by localised cooling. However, the blockade of both TRPA1 and TRPM8 by simultaneously administering the two antagonists to the same mouse, significantly inhibited the vasoconstriction induced by the cold probe (Fig. 2e). This suggests that under localised cooling, both TRPA1 and TRPM8 channels are required to produce a physiological response of vasoconstriction, as vascular response was prevented only when both channels were blocked together. The result provides an insight into the signalling complexities of thermo-sensors where they could potentially work together in a synergistic manner depending on the type of cold stimulus.

## Discussion

This study shows the role of “cool/cold” TRP channels, TRPA1 and TRPM8, in vascular response caused by two different cold techniques. As mentioned earlier, TRPA1 has extensively been shown to be a cold sensor, however, understanding its role in the vascular response to cold is still at an early stage. Previously, we have shown that TRPA1 is central to the vascular response produced by cold water immersion [5]. In this study, we confirm that phenomenon and further show that although TRPA1 plays a role in vascular response induced by generalised cooling, it does not influence the vascular response in the setting of localised cooling caused by a cold copper probe. This suggests that other thermo-sensors may be involved in each response and that the probe acts via a TRPA1-independent mechanism. It is important to note that the probe cools a smaller area of the skin than water immersion, and hence, the cold stimulus may not be strong enough to reach the activation threshold of TRPA1, thus, explaining the lack of effect of HC030031. Similarly, since water immersion technique involves whole paw cooling, this might activate more TRPA1 channels than the localised probe touch could. We also considered that the lack of effect of the TRPA1 antagonist may be due to the cold probe application. Possibly, although providing a 10 °C temperature at the skin surface, as measured by our temperature sensor, it may not provide sufficient penetration of cold into the small area of the skin to elicit a noxious cold TRPA1-dependent response.

It is widely accepted that both TRPA1 and TRPM8 are thermo-TRP channels that are activated by cold temperatures, where TRPA1 is responsible for sensing noxious painful cold and TRPM8 is responsible for sensing innocuous cool temperature [36]. We therefore investigated the effect of the TRPM8 antagonist AMTB. AMTB was used at a dose that had previously induced a partial but significant inhibition of the water immersion cold response [5]. Here, we found that AMTB again was able to produce a significant inhibition of the cold water induced vascular response; but no inhibition of the localised cold-probe-induced response was observed. The result again suggested that localised cooling-induced vascular response is mediated via thermoreceptor other than the classical cold TRP channels TRPA1 and TRPM8.

The lack of role of TRPA1 and TRPM8 in localised cooling is not surprising, as there is still a big debate in literature on whether TRPA1 and TRPM8 channels are cold sensors. Although there is a plenty of evidence in the literature that shows TRPA1 is a cold sensor, there are also studies which show contradictory findings, with some suggestion of an indirect activation [13]. The first evidence of the role of TRPA1 in cold sensitivity was shown by Story et al. [44] where they showed that Chinese hamster ovary (CHO) cells transfected with TRPA1 were activated by cold. However, other groups were unable to produce similar data in HEK293 cells and oocytes that were transfected with TRPA1 [26, 39]. This may be due to the difference in cell type but Zurborg’s group showed HEK293 cells expressing TRPA1 are indirectly activated by cold [49]. Furthermore, strikingly, dorsal root ganglion (DRG) neurons isolated and cultured from mice showed no correlation between TRPA1 and cold temperature in a study by Munns and colleagues, but others have shown DRG neurons to be cold sensitive via TRPA1 activation [19, 38, 43]. Several reasons have been proposed for the disparities between studies in the literature, such as different cell types, conditions, and methodologies.

Conflicting results, concerning the relative importance of TRPA1 and TRPM8, were also found in vivo [33]. Studies were carried out on two different TRPA1 KO mice strains, but with similar deletions. The results showed that TRPA1 KO mice did not [8] or did mediate cold sensitivity to acetone [32]. When reviewed by Kwan and Corey (2009), they pointed out that the precise nature of the experimental procedures performed to detect noxious pain may have been responsible for the contradicting results and that TRPA1 is a noxious cold sensor [33]. Overall, it is realised that TRPA1 can detect noxious cold, although gender and genetic influences may be influential, especially if the cold sensitivity is of short duration [14, 30]. We have suggested that some of the in vivo controversy from the studies in mice may have been due to blood flow changes affecting sensation of pain in response to noxious cold, in a manner that was not at the time realised until our publication that TRPA1 is a vascular cold sensor [5]. Thus, in terms of published results, it is not unusual to find conflicting reports of TRPA1 knockout mice or antagonists having different results in similar systems. Nevertheless, it was surprising that neither the TRPA1 antagonist nor the TRPM8 antagonist had effects in modulating the blood flow response induced by the localised cooling of the mouse paw in our study.

The cold probe is composed of copper which allows it to act as an excellent thermal conductor, (400 k). Thermal conductivity [k] is the property of a material to conduct heat; heat transfer occurs at a lower rate across materials of low thermal conductivity than across materials of high thermal conductivity. In the International System Units (SI), thermal conductivity [*k*] = Watts/meter × Kelvin (or °C). The higher the value of *k*, the higher the property to conduct heat. According to Altshuler and co-workers (1999), the initial temperature of a basal layer skin (pork) at 30 °C decreases to 20 °C in 2 s when put in contact with liquid water at 5 °C, whereas the same skin temperature is reduced from 30 to 12 °C when in contact with copper surface at 5 °C [1]. This is related to thermal conductivity differences, which is almost 700 times higher between copper (400 k) and liquid water (0.6 k). This has the potential to mediate a different effect, in that the skin may lose heat more rapidly, when in contact with copper than water in the same temperature [1]. Thus, an alternative possibility is that the vascular response is initiated by unknown cold sensors. Intriguingly, other cold-sensing mechanisms have been suggested that are independent of TRPA1 and TRPM8 [38]. Munns and colleagues showed that cooling activates a component of superior cervical ganglion neurons, which did not respond to either the TRPA1 agonist mustard oil or the TRPM8 agonist menthol. Thus, we may be at an early stage of elucidating the detailed mechanisms by which the body responds to cold.

It has been recently suggested that TRPA1 and TRPM8 function synergistically in detecting innocuous and noxious cold [47]. Using TRPM8 KO and TRPA1/TRPM8 double KO mice, the authors showed that the double knockout mice showed a large reduction in cold avoidance compared to that of TRPM8 knockout mice, and suggested that both receptors work together to detect the entire cold temperature range [47]. Indeed, in our previous study involving cold water immersion in genetically modified mice with and without the TRP antagonists, we had concluded that TRPA1 and TRPM8 could act via separate but distinct mechanisms to modulate the cold vascular response [5]. Here, co-treatment with the TRPA1 and TRPM8 antagonists significantly blocked the cold probe-induced vascular response, suggesting that TRPA1 and TRPM8 work in a synergistic manner to mediate vasoconstriction. This further supports the findings of Winter and colleagues that the receptors can collude to influence the vascular cold response over the entire cold temperature range, resulting, in this case, in a synergistic effect [47].

To conclude, we have studied a localised cold-induced vascular response in the anaesthetised mouse involving a cold copper probe. The localised cooling could not be inhibited by selective doses of the TRPA1 or TRPM8 antagonist. However, the simultaneous administration of both antagonists caused a significant inhibition of the vasoconstriction, which we assessed as decreased blood flow. On the other hand, it remains possible that further cold-sensing receptors in this model are as yet undiscovered, which highlights the need for further work in the field of cold-induced vascular response.
